# Implementation of Multimodal Stimulation and Physical Therapy in Improving the Level of Consciousness and Recovery in Acute Disseminated Encephalomyelitis

**DOI:** 10.7759/cureus.51217

**Published:** 2023-12-28

**Authors:** Harsh R Nathani, Nishigandha P Deodhe, Ruchika J Zade, Grisha R Ratnani

**Affiliations:** 1 Neurophysiotherapy, Ravi Nair Physiotherapy College, Datta Meghe Institute of Higher Education and Research, Wardha, IND

**Keywords:** glasgow coma scale (gcs), coma recovery scale-revised (crs-r), extended glasgow outcome scale (gos-e), multimodal sensory stimulation, acute disseminated encephalomyelitis

## Abstract

This case report aims to explore the use of multimodal sensory stimulation and physical therapy in rehabilitating a 30-year-old female patient with severe acute disseminated encephalomyelitis (ADEM). ADEM, characterized by autoimmune demyelination in the central nervous system, presents challenges in clinical management, particularly in cases with severe motor deficits and coordination issues. The patient's progress was measured using the Glasgow Coma Scale (GCS), Extended Glasgow Outcome Scale (GOS-E), and Coma Recovery Scale-Revised (CRS-R). The patient showed significant improvement in consciousness levels, functional status, and cognitive and neurological function. The study concludes that a collaborative approach involving both therapeutic modalities and active family participation contributed positively to the patient's recovery.

## Introduction

Acute disseminated encephalomyelitis (ADEM), also referred to as postinfectious encephalomyelitis, is a rapidly progressing autoimmune condition that causes demyelination in the central nervous system (CNS), which includes the brain and spinal cord. This demyelination results from inflammatory reactions triggered in response to antecedent infections or immunization events [1]. A century ago, the medical community identified and characterized ADEM, a multifocal inflammatory demyelinating disorder affecting the CNS. Nevertheless, it was in the early 20th century that a comprehensive understanding of this condition emerged. ADEM is categorized into two primary types, which are post-vaccination (ADEM) and post-infectious (ADEM); post-vaccination (ADEM), initially termed "neuroparalytic accidents," was documented in individuals who received Jenner's smallpox vaccine (actually cowpox) following its widespread adoption in 1853, as well as in those who received Pasteur's rabies vaccine in 1885. However, it was not until the 1920s that ADEM gained broader recognition within the medical community. For post-infectious ADEM, in the context of identifiable post-infectious ADEM, it is important to note that antecedent (or concurrent) infection with the measles virus ranks among the most common triggers. About one case of ADEM for every 10,000 cases of varicella-zoster and one case for every 20,000 cases of rubella virus infections, respectively, are reported. In addition, Johnson's 1996 research has documented several other infectious (or parainfectious) factors, including mycoplasma (as well as other atypical pulmonary infections), herpesviruses, leptospira, and borrelia [2].

The prevalence of ADEM stemming from various etiologies has been documented to range between 0.4 and 0.8 cases per 100,000 individuals in the population [3]. In pediatric research, it has been observed that the median age at which the onset of ADEM typically occurs ranges from 4.5 to 7.5 years of age [4]. In an investigation involving adult patients, it was found that the median age of onset for ADEM was recorded as 33.5 years [5]. Systemic manifestations, such as fever (occurring in 43% to 52% of cases), headache (45% to 58% of cases), malaise, and myalgias, may manifest shortly preceding the onset of neurological signs and symptoms [6]. While motor deficits are observed in both adult and pediatric populations, sensory impairments are more prevalent among adults, whereas seizures are more predominant in pediatric cases. A particular study has documented that approximately 70% of children experience prolonged focal motor seizures, often progressing to status epilepticus. In childhood cases of ADEM, peripheral nervous system involvement is infrequent, but in adults, it tends to be more common, typically presenting as acute polyradiculoneuropathy [5]. ADEM is an infrequent demyelinating disorder, frequently post-viral in origin, and predominantly prevalent among pediatric patients as opposed to adults. From a clinical perspective, it exhibits a variety of manifestations, typically leading to encephalopathy and multifaceted neurological deficits.

ADEM typically initiates with early symptoms, such as fever, nausea, vomiting, headaches, and meningism. Moreover, it invariably encompasses encephalopathy, as per its defining characteristics, often presenting as drowsiness and confusion. Magnetic resonance imaging (MRI) serves as the diagnostic modality for ADEM. It reveals asymmetrical, widespread, and tumefactive lesions, which can be situated both above and below the tentorium cerebelli [7]. In the deeper areas of white matter and at the boundary between gray and white matter, it often appears as hyperintensities on fluid-attenuated inversion recovery (FLAIR) sequences on MRI [8]. ADEM presents with a variety of clinical presentations, including weeks of progressive improvement with varying complete recovery rates (50-70%). Subacute visual impairments may be brought on by lesions affecting the visual cortex and bilateral optic neuritis, which may ultimately result in cortical blindness. A case study that was recently presented demonstrated tetraplegia with bilateral vision impairment, which was most likely caused by the involvement of the occipital lobe and subcortical white matter tract. A comprehensive strategy that includes replacement, compensation, and visual restoration therapy is necessary for the rehabilitation of these patients. Moreover, this approach requires adjustments to everyday living and home environments. Although secure ambulation can be facilitated by using a white cane and auditory cues, sensorimotor and bladder dysfunctions are complex due to cortical blindness, and management requires an extensive multidisciplinary rehabilitation program [9].

For those who have suffered a severe ADEM incident, physical therapy is essential to their recovery, particularly if they are experiencing difficulties with their motor function and coordination. The goals of this therapy are to improve muscle strength, balance, and gait. Notably, there are no known contraindications for physical treatment in this situation. Muscle strength can be improved with exercise, especially in individuals who are temporarily losing their motor abilities. Nevertheless, it is crucial to remember that while creating an exercise regimen, each patient's age and specific needs should be considered. This may require guidance from a physiotherapist or personal trainer [10]. The physical treatment regimen used with individuals who have encephalomyelitis consists of an organized exercise program. It starts with muscular facilitation and relaxation methods, focusing on the lower leg area with the Bobath approach. Here, reducing spasticity and promoting lower extremity mobility are the main goals. The treatment then concentrates on improving the stability of the core by engaging the deep trunk muscles.

Exercises, such as push-ups and sit-ups, are parts of this phase, as is correcting your posture when standing and sitting. In addition, the program includes progressive mobility exercises, which include rotations from right to left, lying to sitting, sitting to standing, and finally reaching independent standing. When combined, these activities offer a thorough strategy for assisting patients with encephalomyelitis in achieving a functional recovery [11]. Patients in a minimally conscious state (MCS) may respond more behaviorally to a sensory stimulation program, even if it would not be enough to bring them back to full consciousness on its own. A program of multisensory stimulation that includes auditory, visual, tactile, olfactory, and gustatory stimuli may be able to enhance patients' recovery when combined with other well-established treatment techniques, such as amantadine [12].

## Case presentation

Patient information 

The patient is a 30-year-old female who was brought to the casualty with specific symptoms of altered sensorium, reduced appetite, generalized weakness and vomiting, and no known underlying health conditions. She has experienced altered sensorium since one day. In addition, she reported a decreased appetite that has persisted for three days. The patient has also complained of generalized weakness affecting both her upper and lower limbs, with this symptom emerging within the last day. Furthermore, she has been suffering from vomiting episodes occurring shortly after food intake, a concern that has been present for the past day, for which she approached our tertiary care for additional treatment. Crucially, the patient has no history of hypertension, diabetes, or any other comorbidities. Moreover, there is no record of head injury, fever, seizures, loss of consciousness, or headaches in her medical history. Here, she underwent management for eight days with medications given, including Niftas, Levipil, Pan, Neurobion, and Modalast tablets. 

Clinical findings

Upon the initial departmental visit, consent was obtained from the patient's relative before proceeding with the examination. The patient's somatic characteristics aligned with an ectomorphic build, showcasing normal vital signs. Notably, both the upper and lower limbs exhibited tense and shiny overlying skin, with a diffuse swelling distally. Upon evaluation, the patient's Glasgow Coma Scale (GCS) score indicated severe impairment (E1VTM1). The patient was in a comatose state. During the examination, the patient was positioned supine with the head end elevated at a 30° angle. Notably, the patient was under mechanical ventilator support in a continuous positive airway pressure (CPAP) mode, with a fraction of inspired oxygen (FiO_2_) set at 60% and a positive end expiratory pressure (PEEP) of 5 cmH_2_0. The presence of Ryles and Foley catheters was noted. In terms of sensory assessment, it was not feasible to conduct. By contrast, motor examination revealed the absence of tone (graded as 0 according to the tone grading system) in both the right and left upper and lower limbs. Edema of grade 2 was observed on the bilateral dorsum of the hands and feet. Deep and superficial reflexes were both absent. Functionally, the patient had an inability to roll independently.

Diagnostic assessment

**Figure 1:**
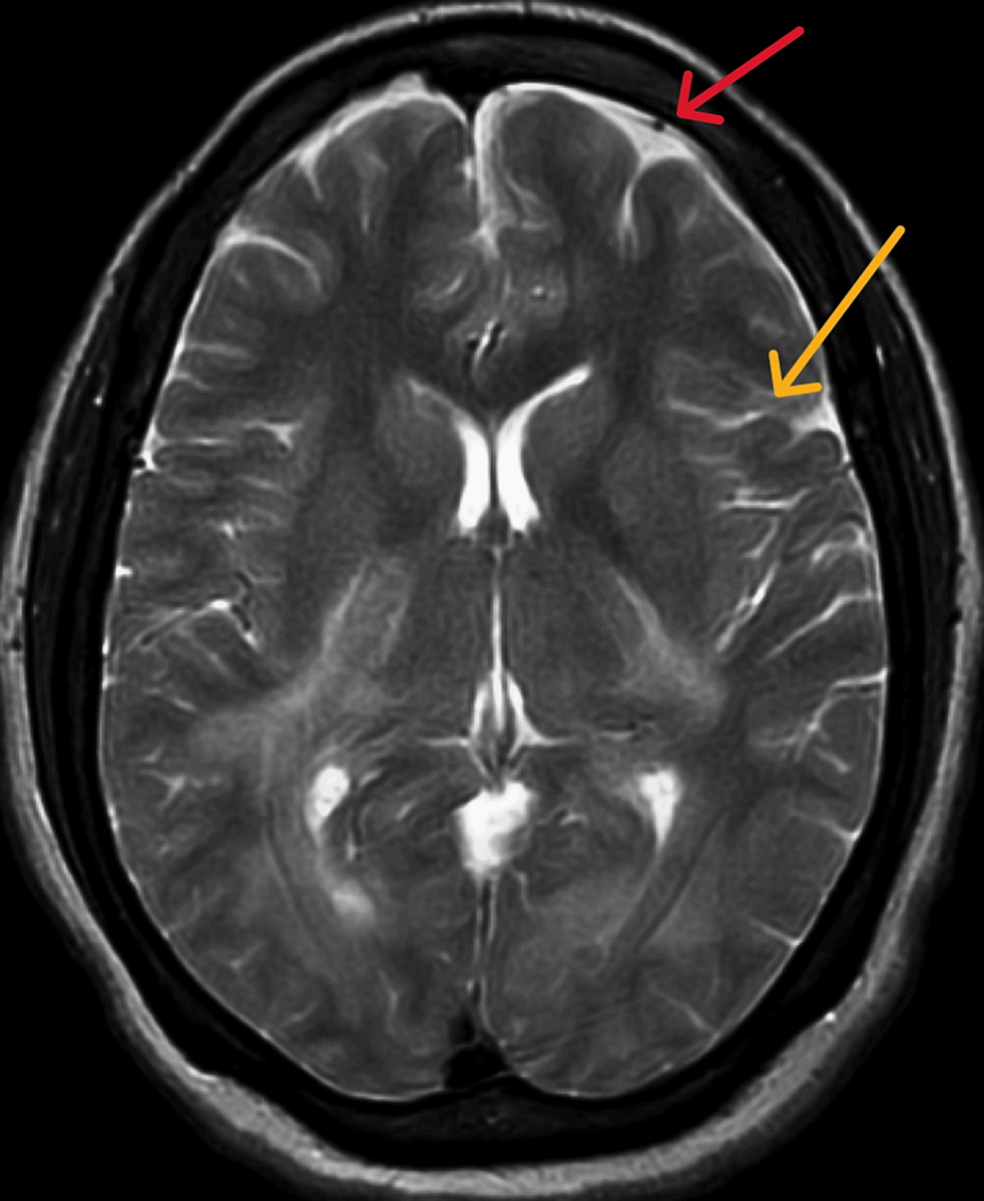
Magnetic resonance imaging (MRI) brain The red arrow denotes dural diffuse, and the yellow arrow denotes leptomeningeal markings.

**Figure 2:**
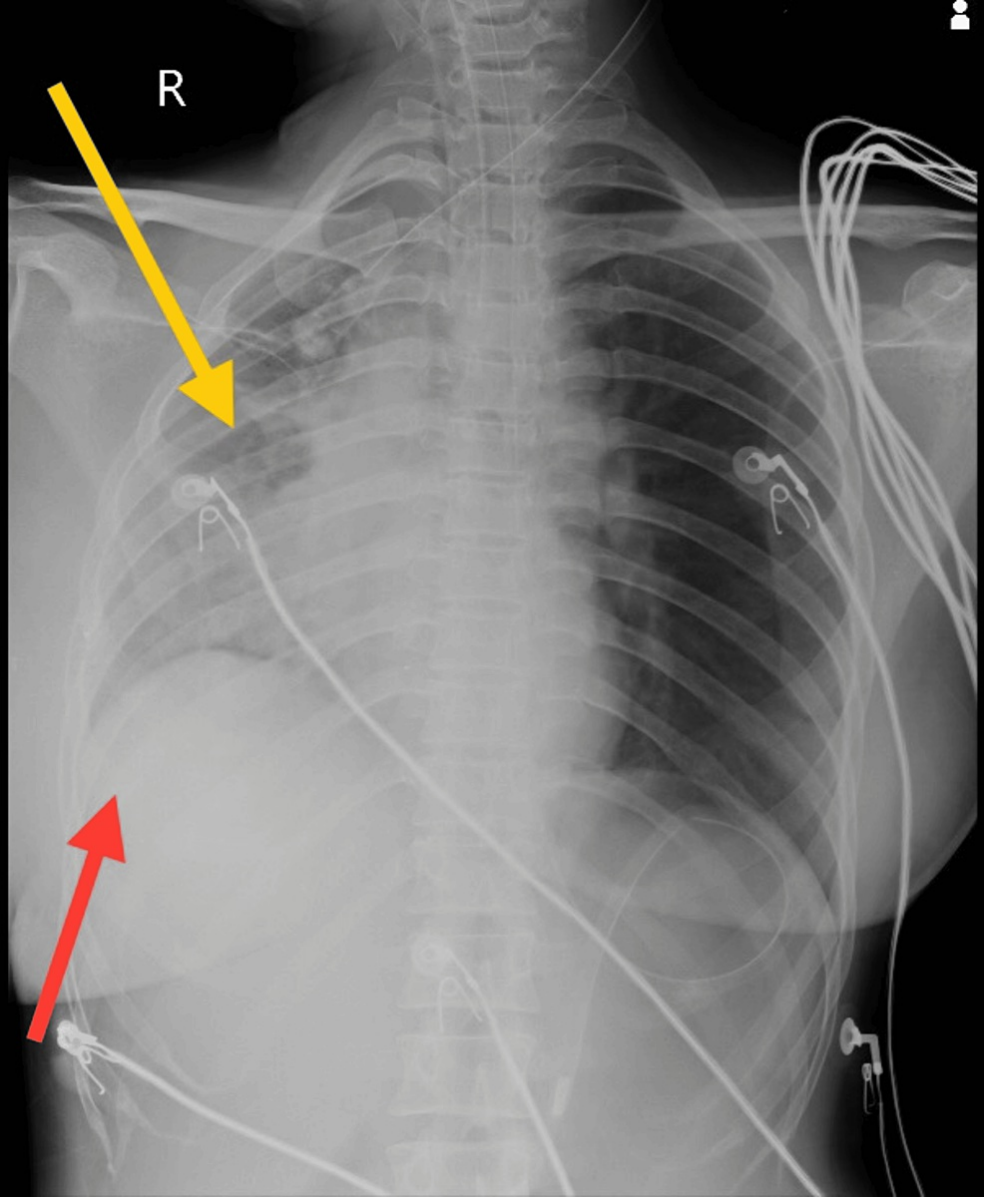
Chest X-ray in the anteroposterior (AP) view Yellow arrow depicts haziness on the right side of the chest with heterogenous opacity. Red arrow depicts elevated diaphragm with blunting of the costophrenic angle.

The arterial blood gas analysis (ABG) report shows the values of pH as 7.292, pCO_2_ as 49.8 mmHg, and HCO_3_ as 21.6, which indicates partially compensated respiratory acidosis. Subsequent tests, encompassing a complete blood count, serum calcium, serum phosphorus, liver and kidney function, and lipid profile, demonstrated results within normal ranges. The computed tomography scan (CT scan) reveals a well-defined, round to oval fluid density lesion measuring 1.1x0.8 cm in the superior sagittal sinus. An electroencephalogram (EEG) recording was abnormal, showing no evidence of ictogenic discharges. The MRI displayed metabolic encephalopathy affecting various regions, including the bilateral corona radiata, bilateral centrum semiovale, posterior limb of bilateral internal capsule, bilateral periventricular region, bilateral fronto-parieto-occipital region, bilateral brachium pontis, brainstem, and left cerebellum, which are mentioned in Figure 1. During bronchoscopy, a thick white mucus plug was observed in the right main bronchus and was aspirated. Subsequent bronchoalveolar lavage revealed the presence of staphylococcal pneumoniae. Lumbar puncture results displayed turbid cerebrospinal fluid and elevated protein levels. The chest X-ray, taken in an anteroposterior (AP) view, revealed an elevated diaphragm, haziness on the right side with heterogenous opacity, a blunt costophrenic angle, and prominent bronchovascular and hilar markings, as shown in Figure 2.

Therapeutic interventions

In the patient's care and interventions, passive range of motion (PROM) exercises involve 10 repetitions for each exercise, with two sets for both bilateral upper and lower limbs, aiming to prevent muscle atrophy and maintain joint mobility in non-ambulatory patients. Rood's facilitatory approach includes 10 repetitions in one set, focusing on a neurological rehabilitation technique to improve muscle tone and coordination. Positioning and wheelchair mobilization are performed for 10 minutes to prevent complications and maintain mobility and independence. The patient was in coma and underwent a tracheostomy and is on mechanical ventilation in synchronized intermittent mandatory ventilation (SIMV). Multimodal sensory stimulation is the treatment that simultaneously stimulates the senses of hearing, sight, touch, smell, and taste. Measurement of consciousness (GCS) and pre-test GCS in the initial evaluation of the her consciousness level was done before undergoing multimodal sensory stimulation.

On post-test GCS, the evaluation of the patient's consciousness was done after receiving multimodal sensory stimulation. The interventional modalities used encompassed a range of sensory stimuli, namely, auditory, visual, tactile, olfactory, and gustatory stimulation apparatus. Auditory stimulation involved addressing the patient by her name and revising personal reminiscences with her for a five-minute duration, maintaining a 10 cm distance from both ear canals. Family members participated in this stimulation by addressing the patient's name three times and subsequently recounting familial memories involving the patient until the allotted time elapsed. Visual stimulation consisted of displaying family photographs to the patient for a five-minute period, positioned approximately 10 cm away from her eyes. If the patient does not open her eyes during this process, a nurse assisted in reopening them. For five minutes, the patient received tactile stimulation. First, the patient's shoulders were softly massaged by the caregivers, and then the patient's hands were gently shaken and rubbed.

**Figure 3:**
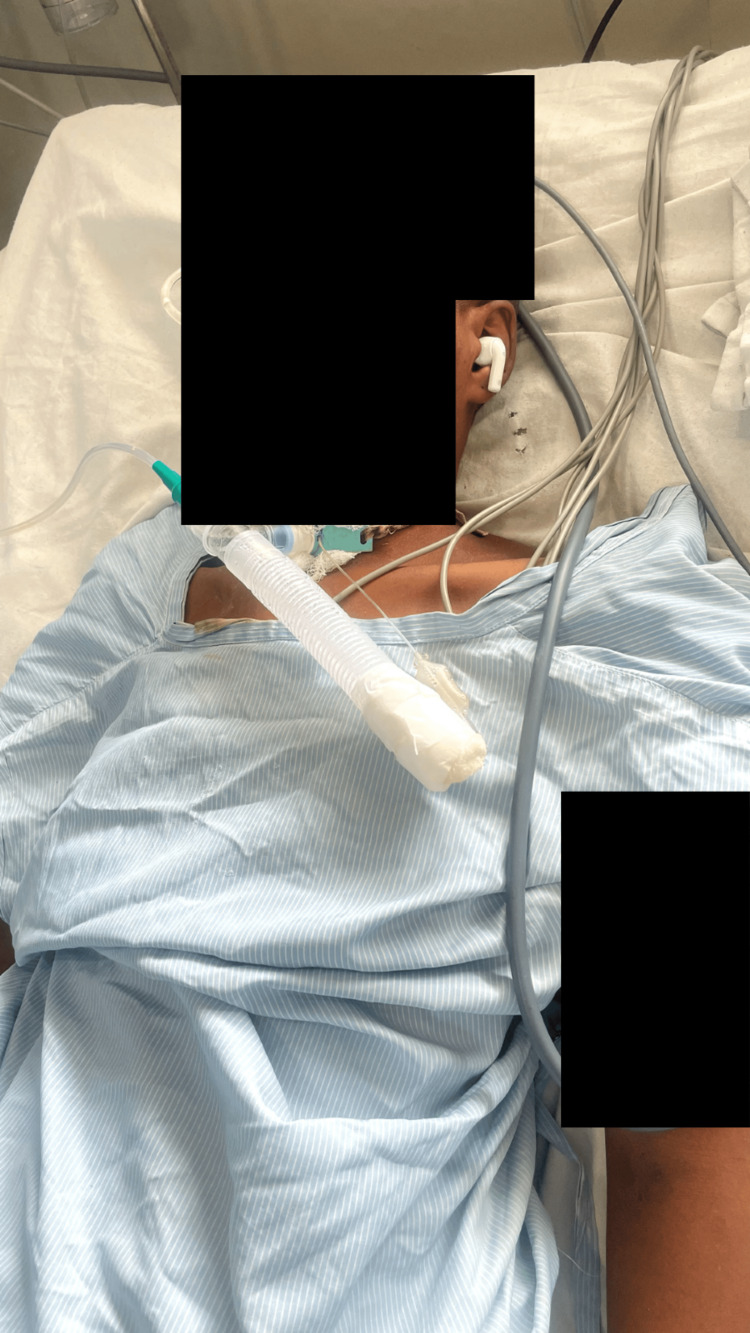
Tactile stimulation via earphones

**Figure 4:**
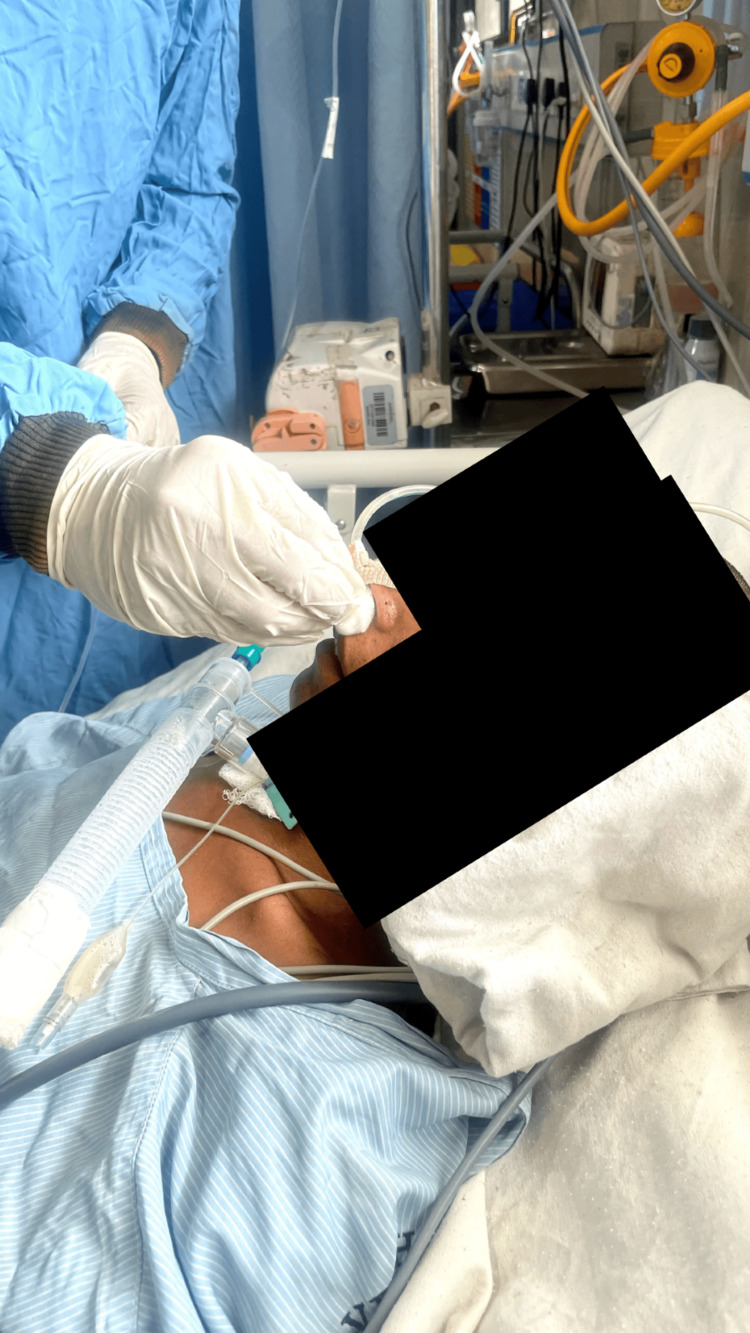
Tactile stimulation using a wisp of cotton

**Figure 5:**
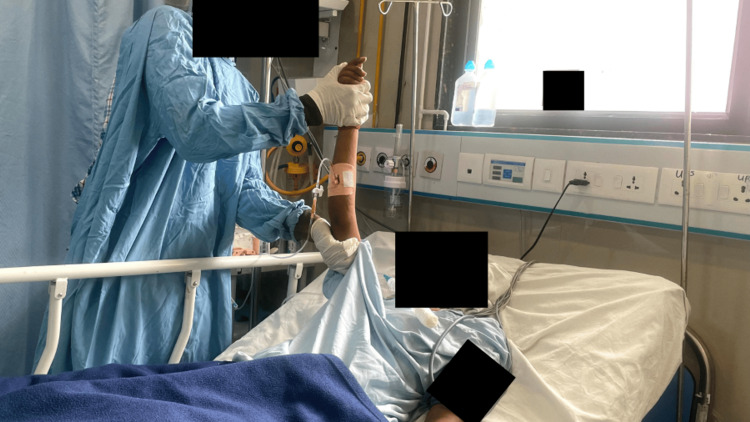
PROM exercises for upper extremity PROM: passive range of motion

Olfactory stimulation was provided for five minutes by presenting scents typically associated with the patient's preferences, situated three cm away from his nostrils. Gustatory stimulation lasted for five minutes and involved applying lemon juice and other different flavours to a cotton swab, which was then applied to the tip of her tongue and above the upper lip. Each stimulation modality was conducted over a five-minute period, resulting in an entire session lasting for half an hour. Multimodal sensory stimulation was administered to subjects five times daily for five consecutive days, with sessions separated by two-hour intervals multimodal intervention, as shown in Figures 3-5. Following the intervention, the patient was evaluated using post-test GCS scores. All the above-mentioned interventions are described in Table 1 [13]. 

**Figure 6:**
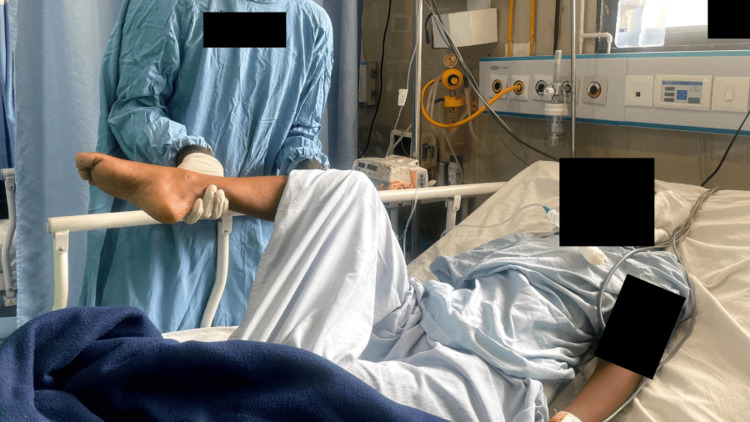
PROM exercises for lower extremity PROM: Passive range of motion

**Table 1:** Therapeutic interventions reps: repetition

Intervention	Strategy	Dosage
To improve the level of consciousness	Multimodal stimulation, visual stimulation, auditory stimulation, tactile stimulation	30-minute sessions, five times a day [13]
To normalize a tone	Proprioceptive neuromuscular facilitation (PNF) (rhythmic initiation) for bilateral upper and lower limb [14] Roods facilitatory approach (icing, tapping, and quick movements) [15]	10 reps, 3 sets and 3 reps, 3 sets
To prevent secondary complication	Positioning	Every 2 hours
To improve speech	Speech therapy	30 minutes thrice a day

Outcome measures 

Following multimodal stimulation administered by the therapist, the patient exhibited notable improvements in various outcome measures. The GCS showed a progression from E1VTM1 to E4VTM5, indicating improved consciousness levels. The Extended Glasgow Outcome Scale (GOS-E) shifted from a vegetative state to a lower level of severe disability, reflecting enhanced functional status. In addition, the Coma Recovery Scale-Revised (CRS-R) score improved substantially, increasing from 5/23 to 11/23, which shows significant progress in cognitive and neurological functions. All outcome measures are shown in Table 2. 

**Table 2:** Outcome measures

Sr No.	Outcome measures	Pre-treatment	Post-treatment
1.	Glasgow Coma Scale (GCS)	E1VTM1	E4VTM5
2.	Extended Glasgow Outcome Scale (GOS-E)	Vegetative state	Lower severe disability
3.	Coma Recovery Scale-Revised (CRS-R)	5/23	11/23

## Discussion

ADEM is a rare, immune-mediated demyelinating disorder of the CNS. It is predominantly considered to be an autoimmune response triggered by viral infections or vaccinations [16]. While ADEM is more commonly diagnosed in the pediatric population, it can also affect adults, typically between the ages of 30 and 50, with no significant gender predilection [17]. Given its rarity and the growing use of MRI in cases of acute encephalopathy, the incidence of ADEM may increase as more accurate diagnoses are made [18]. The diagnostic cornerstone for ADEM is brain MRI, which reveals characteristic asymmetrical, widespread, tumefactive lesions within the CNS.

Clinical presentations of ADEM can vary significantly, often leading to encephalopathy and a spectrum of neurological deficits [3]. In cases where severe motor deficits or coordination difficulties are present, physical therapy becomes an essential component of the management plan [10]. The physical therapy program typically consists of strengthening exercises, facilitation techniques, and active stimulation methods, utilizing the patient's body weight as a training burden. These exercises are implemented in stages, starting with rotational exercises in the supine position, progressing to sitting balance, and ultimately focusing on standing exercises to enhance mobility and independence [11].

Incorporating Rood's approach, sensory stimuli are employed to elicit specific motor responses. This developmental sequence involves progressing from lower to higher levels of motor function with repeated sensory-motor practice until desired outcomes are achieved. Techniques, such as skin stimulation through strokes, moving touch, quick icing, and brushing, are applied to stimulate sensory tracts and facilitate muscle contractions [15].

The core focus of this case report centres on the utilization of multimodal sensory stimulation and physiotherapy in the rehabilitation of a patient with ADEM. Multimodal sensory stimulation, involving auditory, visual, tactile, olfactory, and gustatory stimuli, is recognized as a valuable adjunct to traditional therapeutic approaches. Interestingly, it has demonstrated potential for raising unconscious patients' GCS scores, a measure of improved consciousness. Since multimodal sensory stimulation may be easily included in current therapies, especially for patients with reduced awareness, the family's active participation in giving it is essential.

This collaborative approach not only enhances the patient's sensory experiences but also fosters emotional support from their loved ones [13]. In addition, it aligns with the growing body of evidence suggesting that multimodal sensory stimulation, often accompanied by music therapy, can be effective in comatose and vegetative state patients, particularly after any brain injuries [19]. Multimodal sensory therapy's utility extends beyond ADEM, proving beneficial for individuals in a minimally conscious state or those undergoing physical rehabilitation following various brain injuries. The integration of multimodal sensory stimulation and physiotherapy, along with active family involvement, appears to contribute positively to the patient's recovery. While ADEM presents complex challenges, therapies like multimodal sensory stimulation offer a promising avenue for improving the level of consciousness and overall outcomes in affected individuals [20].

## Conclusions

This case report discusses the management of ADEM in a patient, emphasizing the importance of a holistic approach. The patient's condition is complex, requiring a combination of therapeutic interventions. The report highlights the importance of physiotherapy and multimodal sensory stimulation as integrated components of a multidisciplinary strategy. Physiotherapy focuses on motor function, muscle tone, and coordination, while sensory stimulation engages various senses to enhance sensory experiences. Interventions were chosen based on their individual merits and collective potential to address ADEM's specific challenges. Family involvement in sensory stimulation sessions is a unique aspect of the approach, as it aids in therapeutic delivery and provides emotional support. The reported improvements in consciousness, neurological function, and quality of life validate the rationale behind combining these interventions as a part of a holistic management plan.

## References

[1] Anilkumar AC, Foris LA, Tadi P (2023). Acute disseminated encephalomyelitis. StatPearls [Internet].

[2] Scolding N (2014). Acute disseminated encephalomyelitis and other inflammatory demyelinating variants. Handb Clin Neurol.

[3] Leake JA, Albani S, Kao AS (2004). Acute disseminated encephalomyelitis in childhood: epidemiologic, clinical and laboratory features. Pediatr Infect Dis J.

[4] Gupte G, Stonehouse M, Wassmer E, Coad NAG, Whitehouse WP (2023). Acute disseminated encephalomyelitis: a review of 18 cases in childhood. J Paediatr Child Health.

[5] Schwarz S, Mohr A, Knauth M, Wildemann B, Storch-Hagenlocher B (2001). Acute disseminated encephalomyelitis: a follow-up study of 40 adult patients. Neurology.

[6] Noorbakhsh F, Johnson RT, Emery D, Power C (2023). Acute disseminated encephalomyelitis: clinical and pathogenesis features. Neurol Clin.

[7] Wolska-Krawczyk M (2022). Acute disseminated encephalomyelitis [Article in German]. Radiologe.

[8] Parsons T, Banks S, Bae C, Gelber J, Alahmadi H, Tichauer M (2020). COVID-19-associated acute disseminated encephalomyelitis (ADEM). J Neurol.

[9] Sharma GS, Gupta A, Nashi S, Naveen BP, Khanna M (2021). Rehabilitation in a patient with acute disseminated encephalomyelitis presenting as tetraplegia with cortical blindness. J Neurosci Rural Pract.

[10] Pohl D, Tenembaum S (2012). Treatment of acute disseminated encephalomyelitis. Curr Treat Options Neurol.

[11] Mahayati DS, Aryudha T (2022). Physical therapy in a patient with post-encephalitis tetra-paresis: a case report. Int J Res Med Sci.

[12] Cheng L, Cortese D, Monti MM (2018). Do sensory stimulation programs have an impact on consciousness recovery?. Front Neurol.

[13] Faozi E, Fadlilah S, Dwiyanto Y, Retnaningsih LN, Krisnanto PD, Sumarni Sumarni (2021). Effects of a multimodal sensory stimulation intervention on glasgow coma scale scores in stroke patients with unconsciousness. Korean J Adult Nurs.

[14] Wang JS, Lee SB, Moon SH (2016). The immediate effect of PNF pattern on muscle tone and muscle stiffness in chronic stroke patient. J Phys Ther Sci.

[15] Chaturvedi P, Kalani A (2023). Motor rehabilitation of aphasic stroke patient: the possibility of Rood's approach. Neural Regen Res.

[16] Steiner I, Kennedy PG (2015). Acute disseminated encephalomyelitis: current knowledge and open questions. J Neurovirol.

[17] Pohl D, Alper G, Van Haren K, Kornberg AJ, Lucchinetti CF, Tenembaum S, Belman AL (2016). Acute disseminated encephalomyelitis: Updates on an inflammatory CNS syndrome. Neurology.

[18] Gupte G, Stonehouse M, Wassmer E, Coad NA, Whitehouse WP (2003). Acute disseminated encephalomyelitis: a review of 18 cases in childhood. J Paediatr Child Health.

[19] Kushwaha N, Vyas N, Sidiq M, Gugnani A, Mathur H, Singh J (2023). Effect of multimodal stimulation along with music therapy after traumatic head injury: a case study. HIV Nursing.

[20] Norwood MF, Lakhani A, Watling DP, Marsh CH, Zeeman H (2022). Efficacy of multimodal sensory therapy in adult acquired brain injury: a systematic review. Neuropsychol Rev.

